# Photodynamic exposure of Rose-Bengal inhibits Tau aggregation and modulates cytoskeletal network in neuronal cells

**DOI:** 10.1038/s41598-020-69403-2

**Published:** 2020-07-23

**Authors:** Tushar Dubey, Nalini Vijay Gorantla, Kagepura Thammaiah Chandrashekara, Subashchandrabose Chinnathambi

**Affiliations:** 1 Neurobiology Group, Division of Biochemical Sciences, CSIR-National Chemical Laboratory (CSIR-NCL), Dr. Homi Bhabha Road, Pune, 411008 India; 2 Academy of Scientific and Innovative Research (AcSIR), Ghaziabad, 201002 India; 3 Institution of Excellence, Vijnana Bhavan, University of Mysore, Manasagangotri, Mysore, 570006 India

**Keywords:** Biochemistry, Biotechnology, Cell biology, Neuroscience

## Abstract

The intracellular Tau aggregates are known to be associated with Alzheimer’s disease. The inhibition of Tau aggregation is an important strategy for screening of therapeutic molecules in Alzheimer's disease. Several classes of dyes possess a unique property of photo-excitation, which is applied as a therapeutic measure against numerous neurological dysfunctions. Rose Bengal is a Xanthene dye, which has been widely used as a photosensitizer in photodynamic therapy. The aim of this work was to study the protective role of Rose Bengal against Tau aggregation and cytoskeleton modulations. The aggregation inhibition and disaggregation potency of Rose Bengal and photo-excited Rose Bengal were observed by in-vitro fluorescence, circular dichroism, and electron microscopy. Rose Bengal and photo-excited Rose Bengal induce minimal cytotoxicity in neuronal cells. In our studies, we observed that Rose Bengal and photo-excited Rose Bengal modulate the cytoskeleton network of actin and tubulin. The immunofluorescence studies showed the increased filopodia structures after photo-excited Rose Bengal treatment. Furthermore, Rose Bengal treatment increases the connections between the cells. Rose Bengal and photo-excited Rose Bengal treatment-induced actin-rich podosome-like structures associated with cell membranes. The in-vivo studies on *UAS E-14* Tau mutant *Drosophila* suggested that exposure to Rose Bengal and photo-excited Rose Bengal efficiency rescues the behavioural and memory deficit in flies. Thus, the overall results suggest that Rose Bengal could have a therapeutic potency against Tau aggregation.

## Introduction

Alzheimer’s disease (AD), is the severely emerging neurological disorder, which is considered as a principal cause of dementia^1,2^. The aggregates of Tau are being considered as the hallmarks of AD. Tau protein belongs to type II microtubule-associated protein, which are associated with the stabilization of microtubules^3^. Under physiological conditions, Tau assists in maintaining the microtubule integrity while under pathological conditions, Tau detaches from microtubules leading to the formation of intracellular aggregates of Tau^2,4^. The interference of Tau aggregates in various cellular metabolism processes leads to the generation of toxicity ultimately resulting in the generation of the disease state. Several factors including post-translational modifications (PTMs), reactive oxygen species (ROS), neurotoxin stress, hypertension, physical trauma, diabetes, etc., have been reported to induce modulation in Tau structures, which triggers the aggregation of Tau^5,6^. The modulation in cytoskeleton is considered as one of the major cause for neuronal death^7^. In order to investigate molecules against AD, Tau aggregation inhibition has been considered as an important target of these studies. An array of natural and synthetic molecules have been screened for the potency to inhibit Tau aggregation^8^. Natural molecules-like Oleocanthal, Anthraquinones, Nimbin, Salannin, Curcumin and Cinnamaldehyde have been reported to possess the potency of aggregation inhibition^8–11^. Additionally, synthetic molecules as cyanine dyes, methylene blue, congo red, phenyl amines were studied for inhibition of Tau aggregation^12–15^. Moreover, metal complexes and metal nanoparticles are also reported to have potency against Tau aggregation^16,17^. Several model systems have been studied for screening therapeutic molecules, the in-vitro heparin-induced Tau aggregation and transgenic E14 *Drosophila* is widely examined as a model system for Tauopathy^18–20^. Rose Bengal (RB) is a xanthene dye, which is known to possess the property of photo-excitation and hence it has been widely used as a photo-sensitizer^21^. RB is reported to be effective against various bacterial infections and cancerous cells^22,23^. The potency of RB in inhibiting the Amyloid-β aggregation-induced toxicity was reported earlier^24^. Several factors have been reported for microtubule disassembly; the post-translational modification of Tau is one of the major cause resulting in microtubule destabilization. In present work, we focused on studying the efficiency of RB and photo-excited RB (PE-RB) against Tau aggregation. Our studies were based on the in-vitro biochemical and biophysical methods including SDS–PAGE, Thioflavin S (ThS) fluorescence assay, circular dichroism (CD) spectroscopy, and electron microscopy, which were performed to observe the potency of RB against Tau aggregation. Furthermore, the biocompatibility of RB and PE-RB was studied by monitoring the cell viability assays, which include MTT assay. Moreover, the effect of RB on cytoskeleton modulation was studied by immunofluorescence assay. *Drosophila melanogaster* is an ideal system for studying the neurodegeneration. The transgenic *Drosophila* overexpresses Tau in the nervous system which mimics the human Tauopathy. In our work, the in-vivo studies on *UAS E-14 Drosophila* model were conducted for confirming the protective property of RB against Tau-mediated memory and locomotor dysfunction. Several dyes have been reported to be effective as a therapeutic molecule, the aim of the present study was to analyze the potency of RB and PE-RB against Tauopathy.

## Results

### Rose Bengal inhibits in-vitro Tau assembly

**Figure 1:**
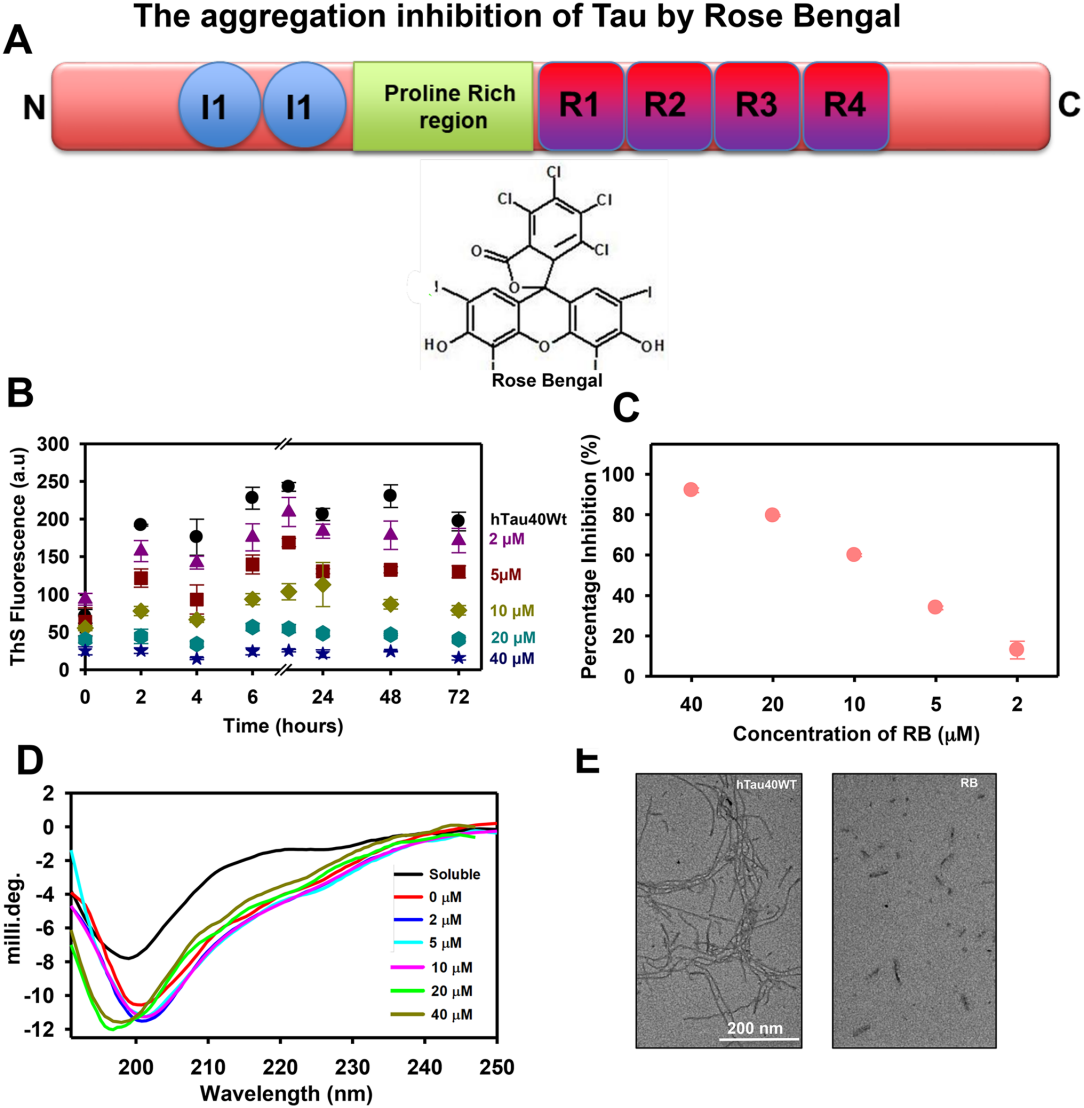
RB inhibits the Tau aggregation in vitro*.* (**A**) The domain organization of Tau. Tau is a natively unfolded protein having two domains, projection domain and a microtubule-binding domain. Rose Bengal is an anionic Xanthene dye widely used in clinical diagnosis. (**B**) The aggregation kinetics demonstrating a reduction in ThS fluorescence in samples incubated with various concentrations of RB. 40 µM RB showed maximum inhibition of Tau aggregation. (**C**) The graph showing the percentage of aggregation inhibition at the end of 72 h. (**D**) CD spectroscopy of RB treated samples, 40 µM RB and 20 µM RB observed to induce conformational changes in Tau aggregates. (**E**) The electron microscopy images of the RB treated sample have small broken fragments of Tau while the untreated sample has long tangled filaments.

Tau is natively unfolded, randomly coiled protein with 441 amino acid in its longest isoform of Tau. The domain organisation of Tau comprises of projection domain, proline-rich domain and microtubule-binding domain. Tau has four repeats in the microtubule-binding domain, which are prone to aggregation^25,26^. Tau protein aggregates and forms paired helical filaments that are considered to be the cause of AD pathology^27,28^. RB is an anionic Xanthene dye, which is applied in various clinical diagnosis purposes (Fig. 1A). The potency of RB in restraining in-vitro Tau aggregation was studied by various biochemical and biophysical methods. For studying the aggregation inhibition potency of RB, recombinant Tau was incubated with heparin and various concentrations of RB (2–40 µM). The results suggested that reduced Thioflavin S (ThS) fluorescence in RB treated Tau, indicating aggregation inhibition (Fig. 1B-C). Tau has a random coil structure in native state whereas, aggregated Tau has characteristic β-sheet. Circular dichroism spectra (CD) of RB treated Tau were analyzed to study the effect of RB on Tau aggregation. The CD spectra analysis suggested that RB induces conformational changes in Tau at concentrations of 20 and 40 µM (Fig. 1D). Furthermore, the electron microscopic analysis was performed to observe the change in aggregates morphology after incubation with RB. These results suggested that RB treatment inhibited the Tau aggregation as small broken filaments were prevalent in the sample. On the contrary, the untreated sample has long, thick filaments (Fig. 1E). The overall studies suggested that RB efficiently inhibits in-vitro Tau aggregation.

### Mature Tau fibrils dissolved by photo-excited Rose Bengal

**Figure 2:**
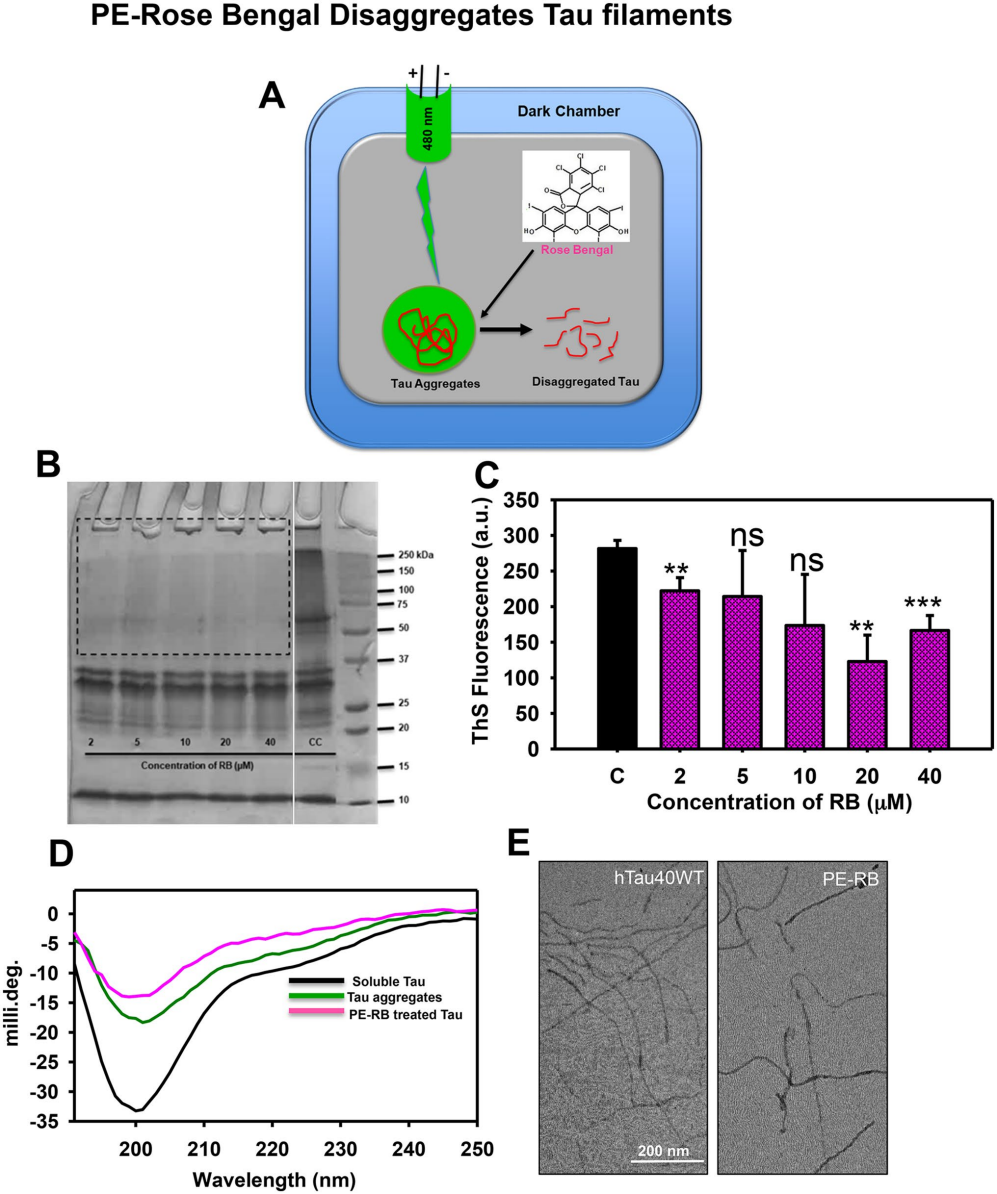
PE-RB disaggregated the mature Tau filaments. (**A**) The schematic diagram demonstrating the irradiation assembly. A dark chamber was designed for irradiating the RB treated Tau aggregates where the green LED was used as a light source. (**B**) The SDS–PAGE analysis of Tau aggregates treated with various concentrations of RB. PE-RB was found to dissolve the aggregates as no higher-order bands were visible in the treated sample compared to untreated control (CC). The gel has been cropped for better representation of results. Raw SDS–PAGE is available with supplementary information. (**C**) The bar graph showing the decrease in ThS fluorescence in PE-RB treated samples indicating an efficient disaggregation of Tau filaments. (**D**) CD spectroscopic analysis of PE-RB treated Tau aggregates showed a shift in random coil region whereas the untreated aggregates have a dip near β-sheet structure. (**E**) The electron microscopy images of PE-RB treated samples showed broken Tau filaments; unlikely, the untreated samples have intact long filaments. p < 0.05, **p < 0.001, ***p < 0.0001 represents the statistical difference between control and treated groups.

RB is one of the well-studied photosensitizers reported to be applied as a therapeutic molecule. The potency of photo-excited RB (PE-RB) was tested against mature Tau filaments. The pre-formed mature Tau aggregates incubated with various concentrations of RB (2–40 µM) were irradiated with green light for 180 min at an irradiance of 9.9 × 10^6^ W/m^2^. The disaggregation potency of PE-RB was addressed by various biochemical and biophysical assays (Fig. 2A). The heterogeneous Tau aggregates could be observed as the higher-order species on SDS–PAGE. Thus, the PE-RB treated aggregates were analyzed by SDS–PAGE for the presence of higher-order species. The SDS–PAGE suggested the dissolution of higher-order aggregates in PE-RB treated samples as a comparison to untreated aggregates where higher-order species were visible (Fig. 2B). Furthermore, the extent of disaggregation was analyzed by ThS fluorescence assay. The reduced fluorescence in PE-RB treated samples in comparison to untreated aggregates suggested a potent disaggregation of Tau fibrils by PE-RB treatment (Fig. 2C). Furthermore, the PE-RB treated aggregates observed to have modulation in secondary structure. The PE-RB treated samples observed to have minimal spectral dip in the random coil region whereas, changes as compared to untreated aggregates (Fig. 2D). Thus, the CD results suggested that PE-RB treatment could break the β-sheet rich aggregates of Tau. The morphological studies including electron microscopy images showed that the untreated Tau aggregated were having long filamentous structure whereas, PE-RB treated samples have broken filament, which evidenced a potent disaggregation (Fig. 2E). Thus, the above-results divulge the disaggregation potency of PE-RB against mature Tau aggregates.

### The biocompatibility of Rose Bengal

**Figure 3:**
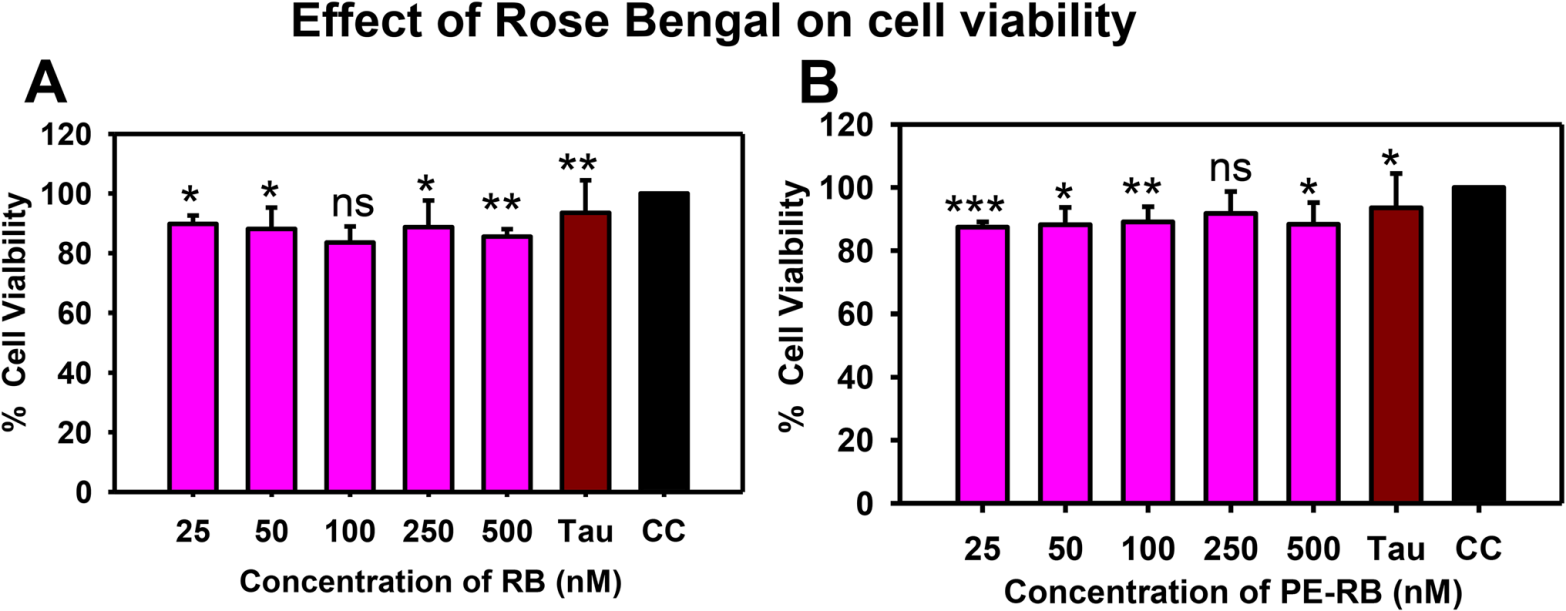
The effect of RB on cell viability. (**A**) The MTT assay performed for observing the effect of various concentrations of RB on cell viability. RB showed minimal cytotoxicity up to 500 irradiation. (**B**) The PE-RB showed negligible cytotoxicity at a concentration of 500 nM. Whereas, the cells treated with 2.5 µM Tau aggregates also showed very minimal reduction in cell viability. p < 0.05, **p < 0.001, ***p < 0.0001 represents the statistical difference between control and treated groups.

The preliminary test to check the biocompatibility of molecule has been investigated based on their effect on cell viability. Thus, to address the therapeutic potency of dye, we aimed to test the effect of various concentrations of RB and PE-RB in Neuro2a cells. The effect of RB and PE-RB on cell viability was observed by MTT assay. Neuro2a cells were incubated with various concentrations of RB. The RB treated cells were irradiated for 10 min at room temperature for photo-excitation. The MTT results indicated that RB and PE-RB showed a minimal detrimental effect on cell viability. An 80% rescue of cell viability was observed even at higher concentrations of RB (Fig. 3A,B). There was minimal cytotoxicity observed after the exposure of Tau aggregates. Hence, due to the generation of low cytotoxicity, RB and PE-RB could be advocated as biocompatible molecule.

### RB and PE-RB modulate the cytoskeleton

**Figure 4:**
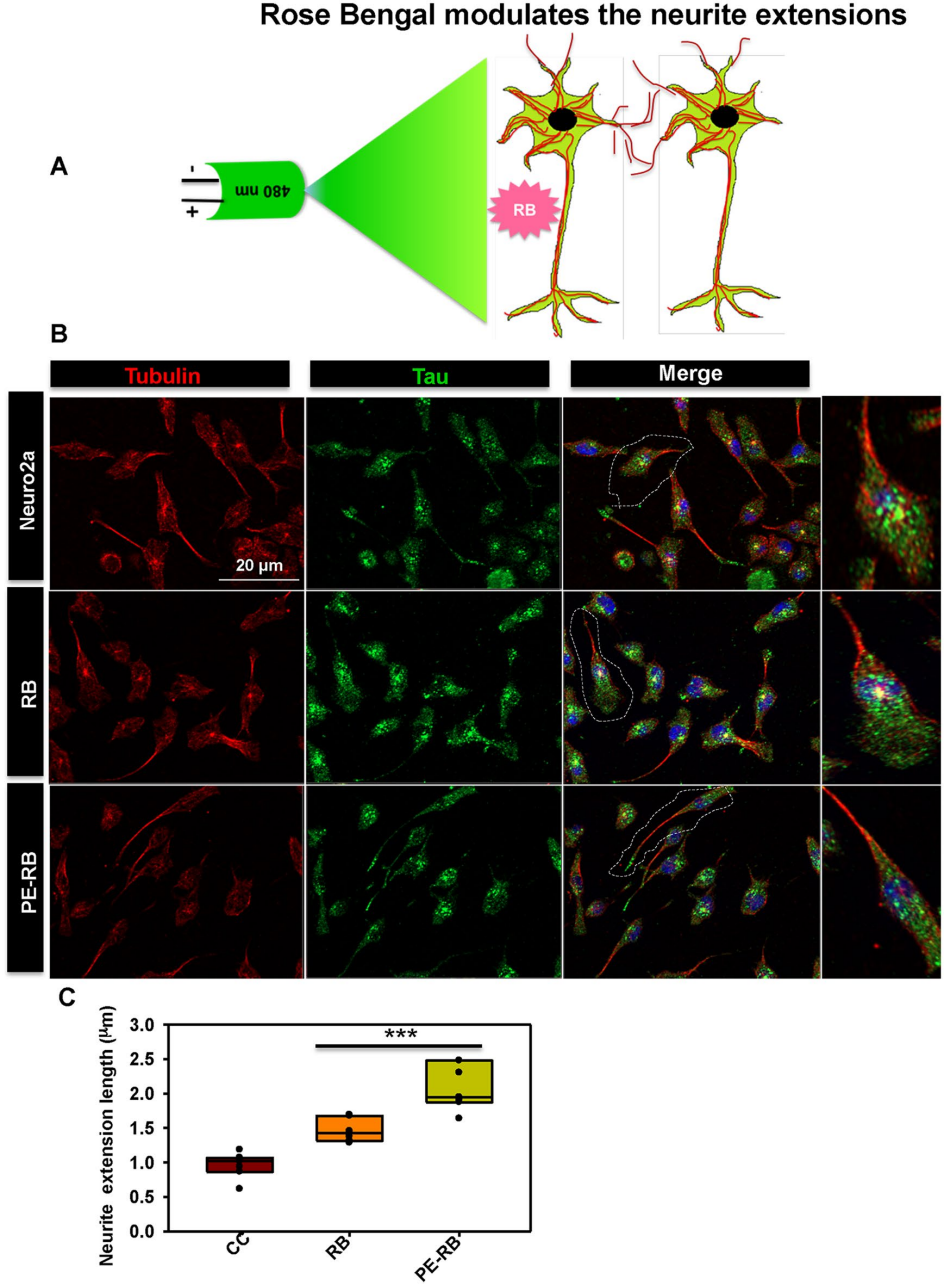
Modulation of the cytoskeleton by RB. (**A**) The effect of RB and PE-RB was monitored on the cytoskeleton network in neuro2a cells. (**B**) The RB and PE-RB treated cells had extended axonal outgrowth with high tubulin intensity. (**C**) The quantification of neurite extension suggested that RB and PE-RB could modulate the tubulin cytoskeleton.

AD has been reported to be associated with various cytoskeletal deformities^29^. Thus, we studied the cytoskeleton modulation in context of RB treatment. Tubulin and actin are the basic cytoskeleton network, which are involved in cell integrity and cell migration^30^. Tubulin has an important role in microtubule stability, cell integrity, cellular trafficking and cell division^31^. The globular actin (G-actin) polymerizes to form filamentous actin (F-actin), which has been reported to be involved in several cellular functions such as cell motility, organelle movement and cell signalling etc.,^32–34^. Actin-rich structures e.g*.* filopodia and lamellipodia are associated with the cell membrane that assist in cell motility, synapse formation and cell adhesion^35,36^. In our work, we aimed to study the effect of RB and PE-RB on the cytoskeleton network (Fig. 4A). Based on the results of cell viability assay, we observe 100 nM of RB as the optimal concentration for cell culture studies, thus further studies were done considering 100 nM of RB. These results suggested that cells exposed to PE-RB treatment were having long neurite extensions in comparison to untreated cells. Although, the modulation in tubulin network was not observed after the treatment (Fig. 4B). Furthermore, we have quantified the length of neurite extensions from the fluorescence images. Here, tubulin was used as a marker of neurite extension. The quantitative data generated using Image J software suggested a significant increase in the length of neurites after the treatment of RB and PE-RB, which indicated the modulation of cytoskeleton by RB and PE-RB (Fig. 4C).

### RB and PE-RB modulates actin network

**Figure 5:**
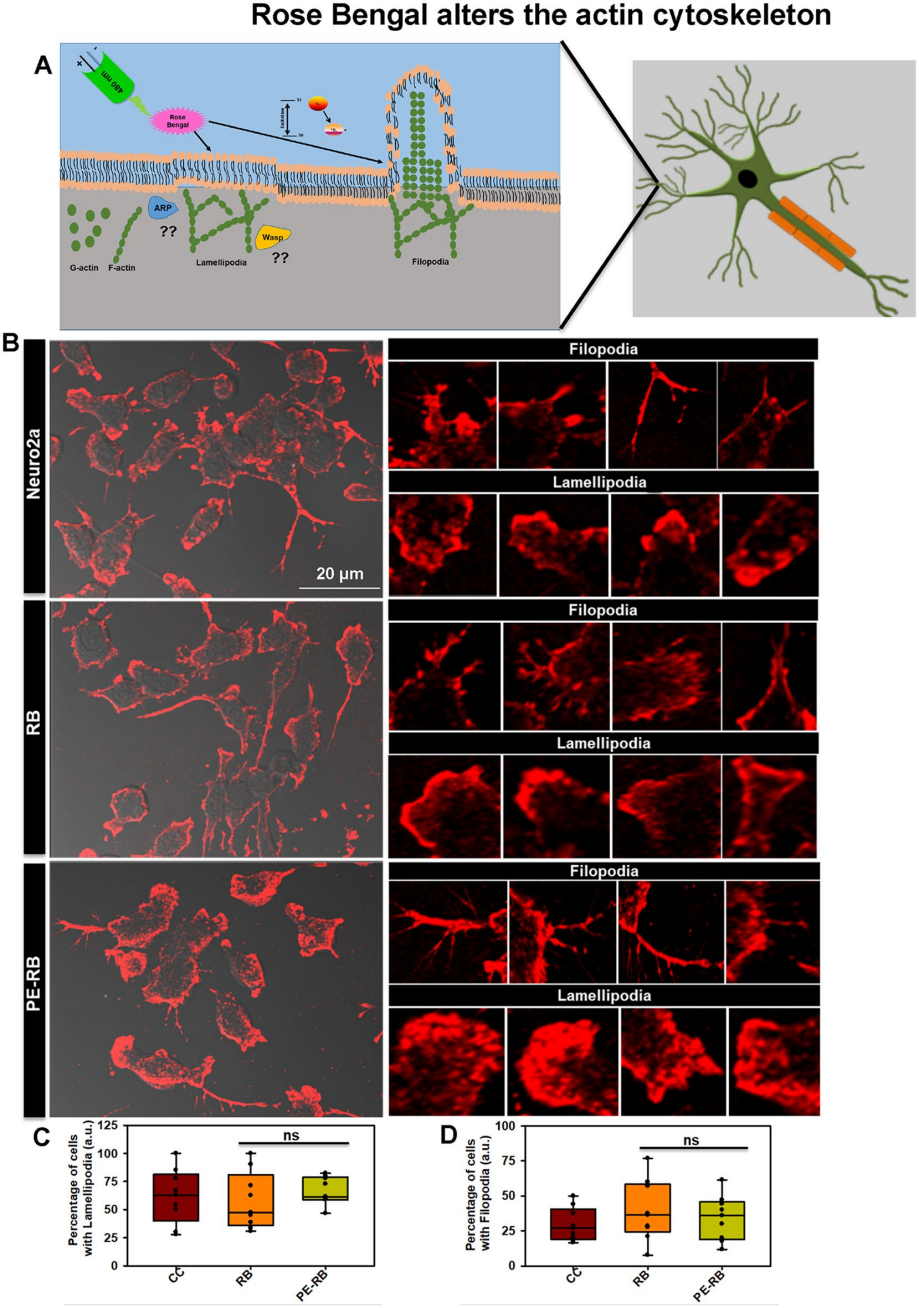
Modulation of the actin cytoskeleton by RB. (**A**) The effect of RB and PE-RB treatment on the actin cytoskeleton was monitored by immunofluorescence. (**B**) The immunofluorescence studies suggested that RB and PE-RB treatment generates various changes in actin cytoskeleton. (**C**) The quantification of images depicting the percentage of cells bearing lamellipodia in each experiment group. (**D**) The cells were quantified for the presence of filopodia using Image J software. The increased number of filopodia were observed in PE-RB treated cells.

**Figure 6:**
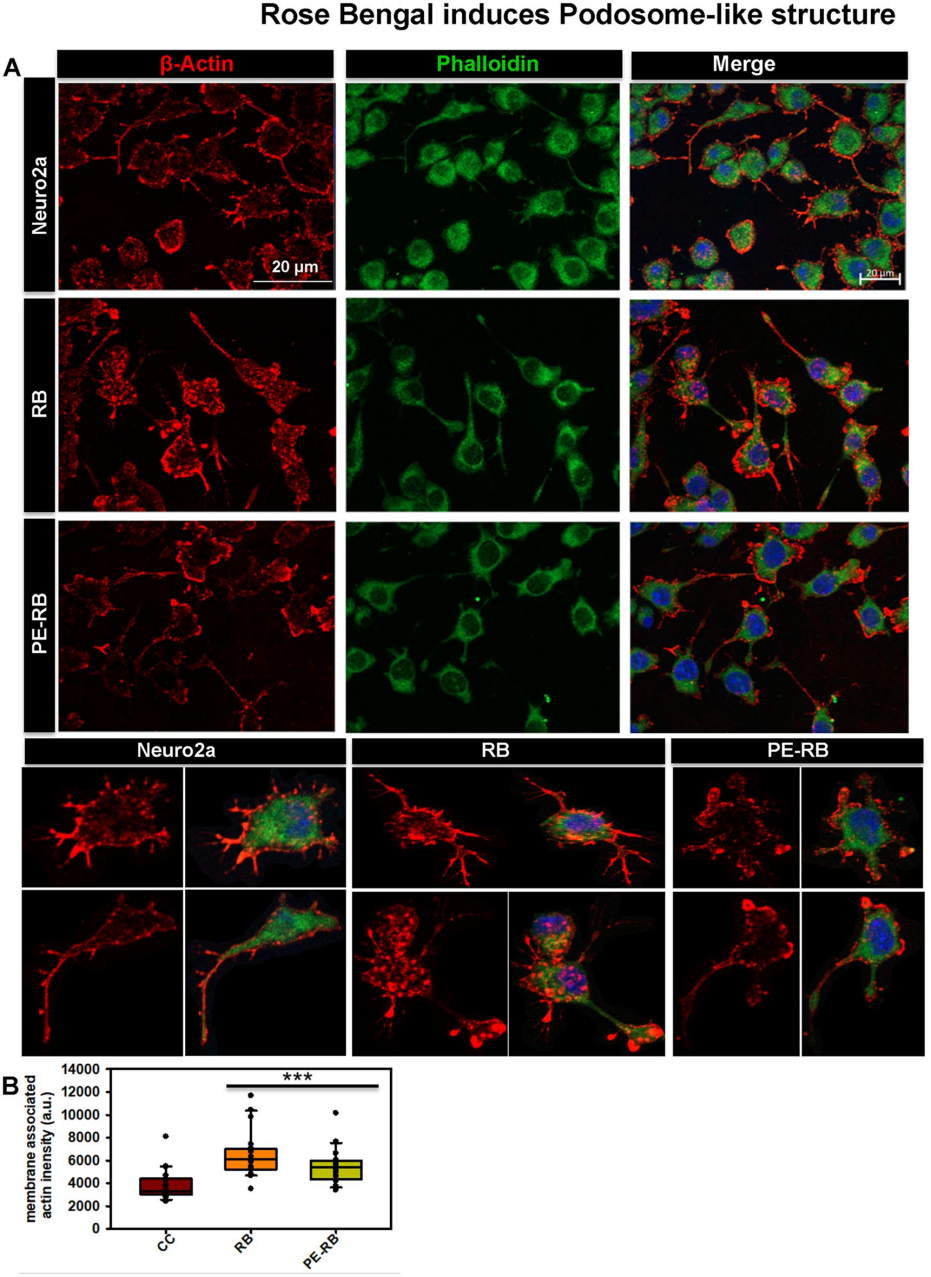
RB and PE-RB increase podosome-like structure in cells. (**A**) The immunofluorescence studies for actin cytoskeleton modulation in presence of RB and PE-RB. The podosome-like structure in the cell membrane were observed after RB and PE-RB treatment. (**B**) The quantification of membrane-associated actin suggesting the increased localization of actin in the membrane after RB and PE-RB treatment.

Actin is a class of cytoskeletal protein that assists in cell motility and migration^33^. Thus, we studied the effect of RB and PE-RB on the actin cytoskeleton. The effect of PE-RB and RB on the modulation of actin modulation was observed by immunofluorescence (Fig. 5A). The cell treated with 100 nM RB showed an increased number of filopodia whereas; lamellipodia structure was not altered as compared to untreated control. Moreover, the increased number of connections were observed in RB treated cells, which was not prominent in untreated cells. PE-RB treated cells were observed to have modulated actin networks, as the cells have prominent growth cone and differentially increased number of filopodia (Fig. 5B). The elevated presence of filopodia structure in RB and PE-RB treatment group suggested that RB has a potency to target the cytoskeleton (Fig. 5C,D). Podosomes are the actin-rich structure, which assists in cell migration. Cell motility and cell adhesion are considered to be the main functions of podosomes. In our work, the cells were treated with 100 nM RB observed to have an increased podosome-like structure in the membrane (Fig. 6A). The quantification of fluorescence images suggested that in PE-RB and RB treatment increased the membrane-associated actin localization, which supported the fact that RB and PE-RB could modulate the cytoskeleton by targeting actin dynamics (Fig. 6B).

### Irradiation time influences cytoskeleton modulation

**Figure 7:**
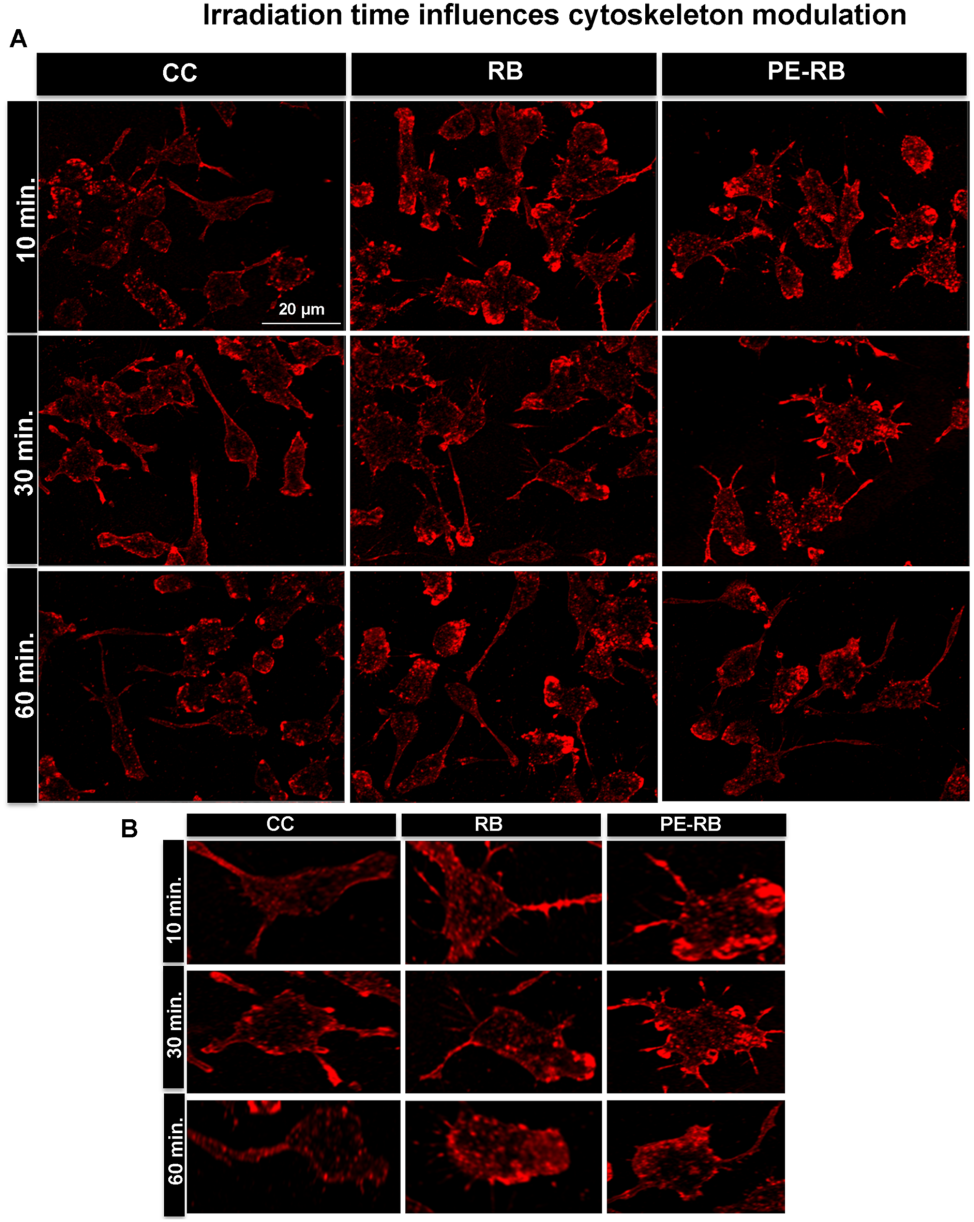
Time of irradiation influences the cytoskeleton. (**A**,**B**) The cells were irradiated for different time points (10, 30 and 60 min). The 10 min and 30 min experimental set showed increased number of filopodia in treated cells. Cells subjected to an irradiation for 60 min showed decreased number of filopodia.

Time-dependent irradiation is one of the factors for deciding the irradiance dose on target. Several studies suggest that irradiance have a role in deciding the potency of photosensitizer^37^. In present work, the effect of varying times of irradiation on actin dynamics was also studied. In our work, 10 and 30 min irradiated cells were observed to possess an increased number of filopodia extensions. On the contrary cells exposed to 60 min of irradiation observed to have reduced filopodia numbers in comparison to cells with shorter irradiation time. The reduced potency of RB upon longer exposure to irradiation could be a result of the photo-bleaching of dye (Fig. 7A,B). Thus, we speculate that RB and PE-RB treatment might accelerate the cell-migration as distinguished filopodial and growth cone modulation was observed after the treatment. Hence, the time-dependent studies suggested that understood that PE-RB and RB potentially modulated the cytoskeleton.

### Protective effect of PE-RB in transgenic *Drosophila*

**Figure 8:**
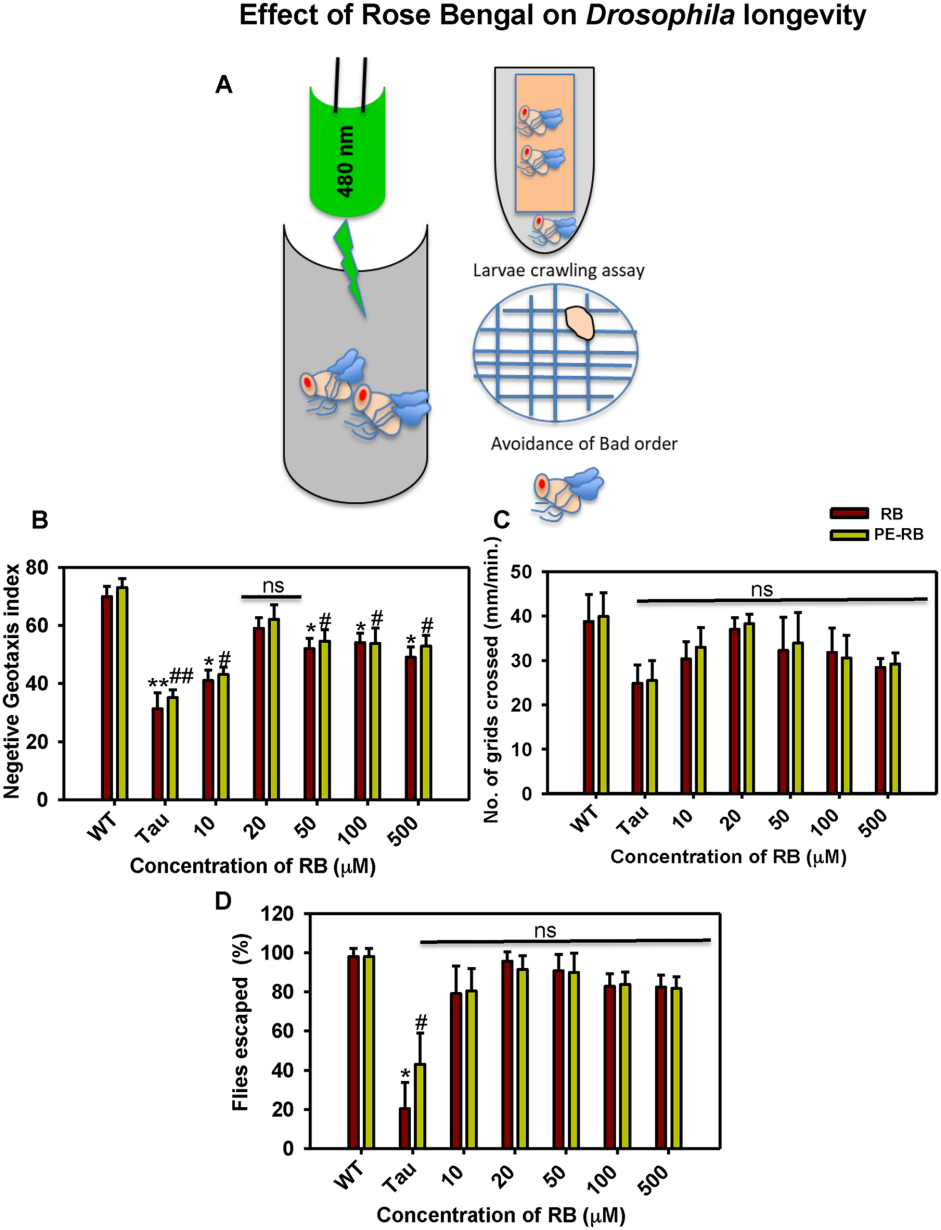
RB and PE-RB rescue the Memory and locomotor deficits. (**A**) UAS E-14 *Drosophila* flies treated with RB and PE-RB, various locomotor and olfactory assay was carried for observing the potency of RB against Tau toxicity. (**B**) A rescue in locomotor function after RB and PE-RB treatment was observed in negative geotaxis assay. (**C**) The larval crawling assay suggesting the positive regulation of locomotor activity by RB and PE-RB treatment. (**D**) Olfactory learning assay suggested that the cognitive function in Tau mutant flies were restored after RB and PE-RB treatment. (*p < 0.05, **p < 0.001, ***p < 0.0001, assigned for the difference between control and treated groups before photo-excitation. ^#^p < 0.05, ^##^p < 0.001, ^###^p < 0.0001 represents the statistical difference between control and treated groups after photo-excitation.).

Tau mutant of *Drosophila* mimics the condition of human Tauopathy by overexpressing the Tau in the central nervous system of *Drosophila*. UAS E14 Tau lines carry 14 disease-associated mutations in Ser-Thr-Pro sites. Many studies have been carried on the *Drosophila* model for screening the molecules against AD^38^. *Drosophila* UAS-E14 flies overexpressing Tau mimics the behavioural aspects of Tauopathy. RB and PE-RB were studied for their potency to rescue the memory and locomotor impairment in mutant *Drosophila* (Fig. 8A). Based on results of in-vitro assay on recombinant Tau, we have studied RB in concentration ranging from 10 to 500 µM. The aim to use the higher concentration of RB was to observe the extent of RB toxicity in the in-vivo model. Negative geotaxis assay allows a rapid, high throughput screening of molecules on a large population of flies. A negative geotaxis assay was performed to study the effect of RB and PE-RB on the locomotory dysfunctions of UAS E-14 *Drosophila*. The flies treated with RB and PE-RB observed to have restored locomotory function. 20 µM of RB and PE-RB observed to be maximum effective whereas, compared to RB, PE-RB found to more efficiency in rescuing the locomotory impairment (Fig. 8B). Succeeding larval crawling experiments were performed for studying locomotory behaviour. In our studies, we noticed that bell-shaped curve in the context of the number of grids crossed by larvae per minute. The larval crawling assay provides precise information for the early locomotor disabilities in *Drosophila,* thus the assay is being performed for screening of various therapeutic molecules. In our studies, 20 µM of PE-RB efficiently restored the locomotory activity of larvae (Fig. 8C). Furthermore, the flies were tested for olfactory dysfunctions in order to study the protective effect of RB and PE-RB on the memory dysfunction. The flies exposed to RB and PE-RB were observed to be efficient in avoiding the bad odour of quinine as compared to untreated mutants. 20 µM of RB showed a remarkable rescue of memory deficit in the flies (Fig. 8D). Thus, RB was found to be effective in rescuing Tau-mediated locomotory and memory deficit in flies. Overall results of in-vivo studies suggested that RB and PE-RB have the potency to rescue the Tau-mediated memory and locomotor impairment thus it could be considered as a therapeutic molecule against Tau.

## Discussion

**Figure 9:**
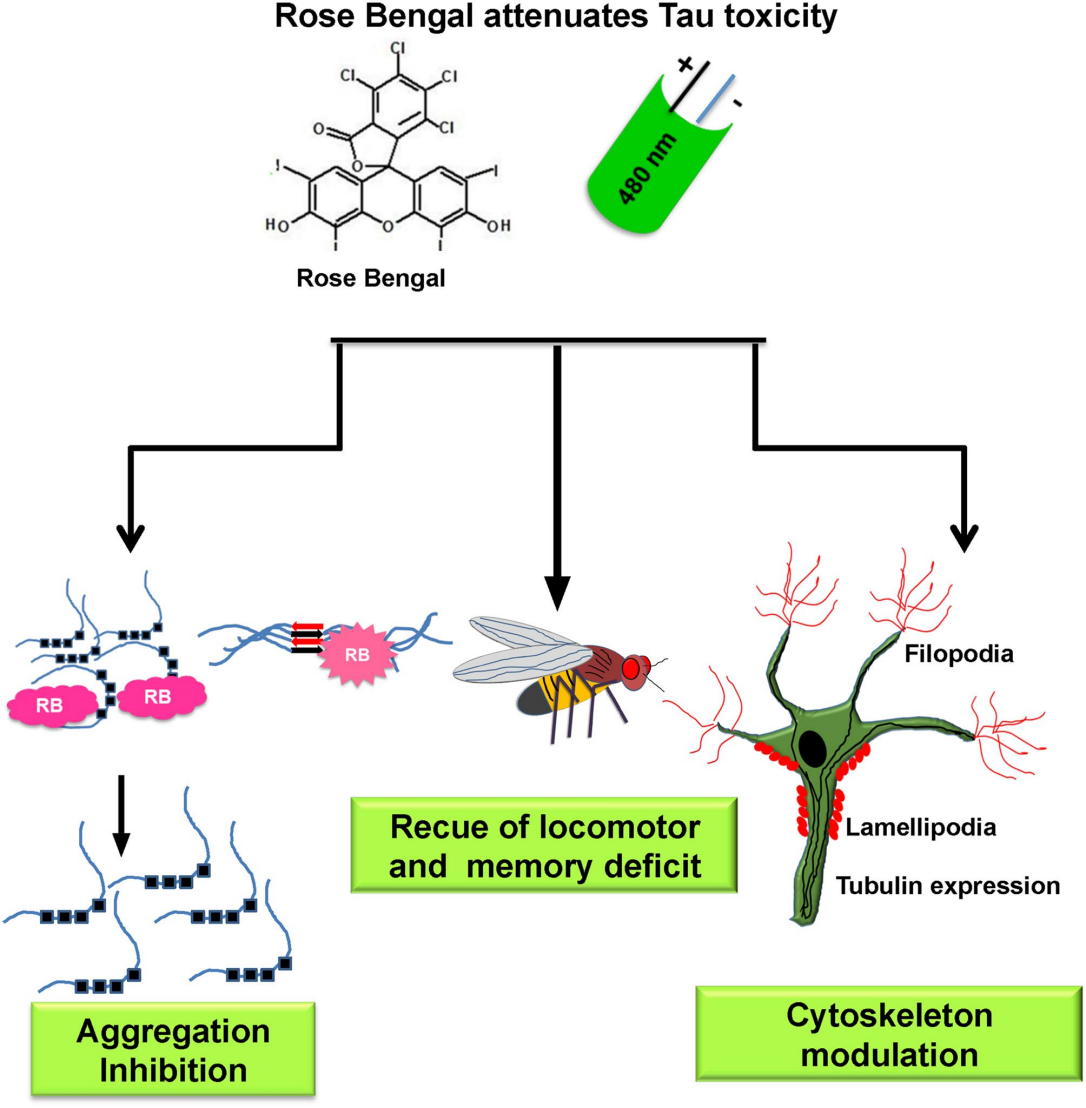
Rose Bengal attenuates the Tau toxicity. The RB attenuates Tau aggregation and photo-excited RB dissolves the Tau fibrils. The RB and PE-RB positively modulate the cytoskeleton. Memory and locomotor dysfunctions of UAS E14 Tau mutants were rescued after the RB and PE-RB treatment. Thus, RB was found effective against the various aspect of Tauopathy supporting it to be a neuroprotective molecule.

AD is prevailing at an exponential rate worldwide, which has emerged as a major cause of dementia^39^. Various aspects of AD have been studied for screening of therapeutics. Intracellular aggregates of microtubule-associated protein Tau is considered to be a leading cause of neurodegeneration in AD^40^. In physiological conditions, Tau is abundantly distributed in axons, which functions to stabilize the microtubules. Furthermore, Tau is being reported to be localized in the nucleus and dendrites, which might function in maintaining the synaptic plasticity and DNA integrity^41^. Accumulation of Tau aggregates alters the viability of neuron in numerous ways as they interfere with signalling cascades, induce ROS and downregulates the cytoskeleton machinery^42^. Different classes of dyes have been investigated as therapeutic molecules in AD. The diazo dye Congo red was reported to attenuate the amyloid-β-related toxicity. Similarly, a cyanine dye N744 was reported to efficiently inhibit the aggregation of Tau^12^. Methylene blue and its derivatives have been studied as Tau aggregation inhibitors^43^. Rose Bengal (RB) is a xanthene dye, which has been widely applied as a photosensitizer in photodynamic treatments^23^. In our studies, RB was investigated for its efficiency against Tau aggregation. RB has been reported to inhibit the fibrilization of another AD-related protein Aβ-42^24^. In present work, RB was observed to be efficient in attenuating the Tau aggregation even at the lower concentrations. In the present study, dual property of RB was observed, as RB inhibited the aggregation of soluble Tau and additionally, the PE-RB dissolved the mature Tau filaments. The biocompatibility of a molecule is among the necessary criteria to be evaluated for a therapeutic molecule. Thus, the toxicity of molecule in neuronal cells have to be considered^44^. In our studies, RB and PE-RB showed low levels of cytotoxicity even at higher concentrations. Microtubules are the basic cytoskeleton elements that assist in maintaining the integrity and structure of cells. Microtubules can be addressed as “polymers” of Tubulin protein as α and β-tubulin polymerizes to generate the microtubules^45^. Cytoskeleton network is a one of the potential for the screening of drugs against various diseases^46^. Several cytoskeleton defects as microtubule destabilization, disturbed actin dynamics and axonal transport are being reported to be related to neurodegeneration^47^. Thus, as a strategy for studying drugs for neurodegeneration, therapeutic molecules have been screened for their potency to revert the cytoskeleton defects^48^. Molecules having the mode of action against the cytoskeleton integrity have been screened as a therapeutics for various diseases^49^. Colchicine, rhizoxin, maytansine, centauriedin, combretastatin, etc.; are the therapeutic molecule, which targets tubulin^50–53^. Photodynamic therapy modulates cytoskeleton assembly in various aspects. The photo-excited porphyrin dye reported to inhibit the tubulin assembly, whereas the xanthene dye RB was observed to disrupt the actin network in cells^54,55^. In our studies also, we observed that RB and PE-RB treatment modulates the cytoskeleton networks of cells. The growth cone and filopodia structure assist the cells in migration, cytokinesis etc^56^. Our results suggests that RB and PE-RB can regulate the filopodia length, which suggested that the treatment might induce accelerated cell motility. Another function of filopodia is to facilitate the synapse formation^57^. Here, we stated that RB and PE-RB treatment assists in the development of connections between the cells, increased filopodia, and actin-rich podosome structures suggested that the RB and PE-RB may induce the modulation in cell migration. Filopodia are actin-rich protrusions that assist the cell in migration and cell motility^58^. AG1478 and cetuximab results in reduced cell migration by inhibiting actin dynamics^59^. Calumenin-15 has been reported to increase filopodia formation by targeting member of TGF-β superfamily ^60^. Similarly, jasplakinolide (Jasp) also induces F-actin polymerization by activating ERK and AKT pathways ^61^. Here based on present study we hypothesized that RB and PE-RB may have potency to modulate various signalling cascades, which are involved in cytoskeleton dynamics. Tubulin is known to play role in neuronal differentiation^62^. The result of our present studies suggested that the PE-RB modulated the tubulin network of cell, thus it could be stated that PE-RB might have potency to induce the neuronal differentiation. *Drosophila* has a small, completely annotated and simple genotype. *Drosophila* has a 69% homology with human genotype, thus for several in-vivo studies, *Drosoph*ila has been chosen as a model organism^63^. UAS E14 Tau is a transgenic flies, which mimics the condition of AD as it overexpresses the Tau protein in nervous system^38^. The screening of therapeutics have been done on the basis of their ability to restore the behavioural and memory deficits in flies^28^. We found that 20 µM RB and PE-RB efficiently restores the memory deficit and locomotor dysfunctions of mutant flies. The cumulative result of all the studies projects PE-RB and RB as a potential therapy against Tau aggregation and hence can be studied for further therapeutic property in AD (Fig. 9).

## Conclusions

Tau aggregation is the major consequence of AD. The antagonist of Tau aggregation could be studied in the aspect of AD therapy. In our study, we observed that RB and PE-RB efficiently inhibit Tau fibrillization. The RB and PE-RB positively modulated the neuronal cytoskeleton. Additionally, in-vivo studies clearly suggested that RB and PE-RB have protective property against memory deficits and locomotor dysfunction in flies. Thus, it could be suggested that RB and PE-RB are effective in in-vitro and in-vivo system against Tauopathy and could be studied as a lead molecule in the treatment of AD.

## Materials and methods

### Chemicals and reagents

MES (M3671), BSA (9048-46-8), BES (10191-18-1), BCA (B9643), CuSO4 (C2284), ThS (T1892), MTT (M2128) and Rose Bengal (330000) were purchased from Sigma. IPTG (420322) and DTT (3870) were purchased from Calbiochem. Other chemicals such as Ampicillin (2007081), NaCl (194848), KCl (194844), Na_2_HPO_4_ (191437), KH_2_PO_4_ (19142), EGTA (194823), MgCl_2_ (191421), PMSF (195381), Ammonium acetate (191404), Heparin (904108) and DMSO were from purchased from MP biomedicals and protease inhibitor cocktail was from Roche (11697498001). Copper coated carbon grids were purchased from Ted Pella (01814.F, carbon type-B, 400 mesh Cu), DMEM advanced F12 media (12634010), Fetal Bovine Serum (16000044), Pensterp cocktail (04693159001) and Anti-anti (15240062) were purchased from Gibco. Tubulin (Thermo PA1-41331), actin (Thermo MA5-15739) were procured from thermo, total Tau antibody K9JA was purchased from Dako (K9JA, Dako A0024). The secondary antibodies Alexa Fluor 488 (A11034) and Alexa Fluor 555 (A32727) were purchased from thermo.

### Preparation of Tau

The recombinant full-length Tau was purified as per published protocol^11^. The recombinant Tau was expressed in *E.coli* BL21* strain. The culture was incubated at 37 °C till OD_600_ reached 0.5 to 0.6. It was then induced with 0.5 mM IPTG and was further incubated for 4 h. The cells were harvested by centrifugation at 4,000 rpm for 10 min. The cells were lysed using Constant Cell Disruption systems. The cells were resuspended in Buffer A composed of 50 mM MES, 1 mM EGTA, 2 mM MgCl_2_, 5 mM DTT, 1 mM PMSF and 50 mM NaCl, this was subjected to homogenization at 15,000 psi pressure. The obtained lysate was heated at 90 °C for 20 min in presence of 0.5 M NaCl and 5 mM DTT. This was cooled and centrifuged at 40,000 rpm for 45 min. The supernatant was collected and dialyzed overnight against Buffer A before loading to the cation exchange column. Tau protein was eluted by applying ionic gradient of 1 M NaCl. The protein quality was analyzed by SDS–PAGE and further subjected to size-exclusion chromatography. The obtained fractions was analysed on SDS–PAGE, pooled and concentrated. The concentration was estimated by BCA assay and stored at − 80 °C until further used.

### In-vitro Tau aggregation

Tau aggregation was induced at 37 °C with heparin as previously described^16^. Tau in the presence of anionic inducer such as heparin, RNA, arachidonic acid, etc. undergoes aggregation. Among all these molecules heparin-induced Tau aggregation is the widely accepted model for in vitro Tauopathy studies. Previous observations suggested the heparin-mediated Tau aggregation demonstrates the transition of Tau from random coil to β-sheets. Taniguchi et al*.* demonstrated the inhibition of heparin-induced Tau filaments by phenothiazine, polyphenols, and porphyrins. In present study Tau aggregation was induced by heparin where the soluble full-length Tau was incubated with heparin (17,500 kDa) at a ratio of 4:1. The reaction was carried out in 20 mM BES buffer supplemented with 25 mM NaCl, 1 mM DTT, 0.01% NaN_3_ and protease inhibitor cocktail mixtures.

### Thioflavin S fluorescence assay

The effect of RB on aggregation of Tau was measured by Thioflavin S (ThS) fluorescence assay^17^. ThS is a mixture of methylated dehydrothiotoluidine and sulfonic acid and has a property to fluoresce to β-sheet structures. The fluorescence measurement was carried out by incubating 2 µM of Tau with ThS at a ratio of 1:4 for 15 min. All the reaction mixtures were measured in triplicate in TECAN Infinite M200 PRO spectrophotometer at an excitation of 440 nm and emission of 521 nm. Further, the data were analyzed using SigmaPlot 10.0.

### Circular dichroism spectroscopy

The conformational changes of Tau were analyzed by using CD spectroscopy in the far-UV region. In native conditions, Tau has typical random coil conformation, but the aggregation causes its conformational change to β-sheet. The effect of RB on conformational changes of Tau was studied as described previously^16^. All the spectra were measured in Jasco J-815 spectrometer by diluting full-length Tau to 3 µM in 50 mM sodium phosphate buffer at pH 6.8 (Supplementary information 1).

### Photodynamic treatment of Tau aggregates

For analyzing the effect of photo-excited RB on Tau aggregates, the aggregates were incubated for 1 h in dark with varying concentrations of RB (2, 5, 10, 20 and 40 µM). 200 µL of the reaction mixture was added in 96 black well plate (Eppendorf) and was irradiated in dark using green LED. After 1 h of incubation, the samples were analyzed by ThS fluorescence assay and SDS–PAGE for the presence of disintegrated Tau. The irradiation dose or irradiance was calculated by the formula

$$E = \frac{{{\text{Power}}}}{{{\text{Area}}}}*{\text{Time of irradiation}}$$

where E is irradiance, time was calculated in terms of seconds**.**

### SDS–PAGE

The inhibitory effect of RB on Tau aggregation was observed by SDS–PAGE. Aggregates are resolved around 250 kDa as higher molecular weight species thus, the effect of RB on aggregation propensity of Tau can be observed by 10% SDS–PAGE. The experiments were done by using BIO-RAD Mini-PROTEAN electrophoresis unit.

### Transmission electron microscopy

The morphological analysis of Tau fibrils were done by electron microscopy and the samples were prepared according to published protocol^17^. For electron microscopic analysis, 2 µM of Tau was incubated on carbon-coated copper grids. Following this, the samples were negatively stained with 2% uranyl acetate. The images were captured by TECNAI T20 at 120 kV.

### Cytotoxicity assay

The cell viability was analyzed by methylthiazolyldiphenyl-tetrazolium bromide (MTT) assay. The method was followed according to published protocol^20^. Neuro2a cells (ATCC CCL-131) were cultured in advanced DMEM F-12 media supplemented with 10% FBS and glutamine. 10,000 cells/well were seeded in 96 well plates for the assays. After 24 h, the cells were treated with various concentrations of RB for 24 h; followed by the addition of MTT at a concentration of 0.5 mg/mL and incubated at 37 °C for 4 h. The formazan crystals formed were dissolved in 100 µL of 100% DMSO. Cell viability was evaluated by measuring the absorbance at 570 nm. Similarly, cells were incubated with 2.5 µM of full-length Tau aggregates to observe their cytotoxicity. Additionally, RB was also added to cells along with aggregates for analyzing its effect of RB in the presence of aggregates. RB treated cells were subjected to 10 min of irradiation for the cytotoxic analysis of photo-excited RB.

### Immunofluorescence

Neuro2a cells were seeded at a density of 25,000 cells on glass coverslips. The cells were treated with 100 nM RB and incubated overnight at 37 °C. Similarly, another set of cells treated with 100 nM of RB were irradiated with green light for 10 min and incubated at 37 °C for 24 h. The cells were fixed with absolute methanol for 20 min at − 20 °C. After fixation, cells were permeabilized by 0.2% Triton X-100 and after 3 subsequent PBS washes the cells were incubated with 5% horse serum for 1 h. Further primary antibodies against tubulin (Thermo PA1-41331), actin (Thermo MA5-15739) and Tau (K9JA, Dako A0024) were added and incubated overnight. The cells were incubated with Alexa Fluor 488 (A11034) and Alexa Fluor 555 (A32727) tagged secondary antibodies. The nucleus was stained with DAPI. The cells were scanned by Zeiss Axio observer 7.0, apotome 2.0 inverted microscope using 63X magnifications in oil emersion.

### Fly stocks and genetics

The transgenic *Drosophila* strain used in this study was UAS-Tau E14. ELAV-Gal4 driver line was obtained from the National Drosophila Stock Centre at the University of Mysore, Mysore, Karnataka, India. *Drosophila* strains were raised on standard medium. Fly cultures and crosses were carried out at 25 °C. The stocks has been maintained as per published protocol^20,64^.

### Fly husbandry

Flies were maintained on standard banana-jaggery medium (SM) under standard laboratory conditions of 24 ± 1 °C temperature, 75 ± 5% relative humidity, and 12:12 light and dark cycle (SLC)^64^. Flies were maintained in a 2 week discrete generation cycle for 10 generations before being used in this study. The adult density was regulated at about 100 flies per half-pint bottle with 25 mL of SM in 10 bottles. Flies from 10 bottles were combined into a single breeding cage, hereafter referred to as parental cage (PC).

### Preparation of diet

A total of 2.5 L of SM was prepared and split into 5 batches of 500 mL each as described previously^28^. For the control group, only SM was poured into the bottles. For the RB-supplemented media, 10, 20, 50,100 and 500 µM of RB was added to SM and mixed thoroughly just before pouring into the bottles. All bottles were plugged with non-adsorbent cotton and the media was allowed to solidify under room temperature.

### Larval feeding behaviour assay

The obtained eggs were transferred at a density of 50-eggs/6 mL of SM and allowed to develop till early third instar(as referred in^20^). The early third instar larvae were removed from the SM vials and used in the feeding behaviour assay. Larvae were individually transferred to an assay Petri plate of 5 cm diameter containing 10 mL of either liquid SM (SM without agar) or liquid SM supplemented with different concentrations of RB and allowed for 5 s for acclimation. The feeding rate was measured as the mean number of sclerite retractions in 2 consecutive 30 s intervals. The average of the 2 rates was taken as the feeding rate of that larva. 20 larvae were assayed for each of the 2 treatment groups. The feeding rate of assays were replicated 4 times. A total of 160 larvae were assayed for the feeding rate.

### Negative geotaxis assay

The ability to move against gravity and climb indicates the level of physical fitness of test animals(as referred in^20^). The vertical climbing ability of male flies that emerged from different treatment bottles were assessed. 20 male flies per treatment group were collected and transferred to empty, 0–15 cm graduated vial. The vial was gently tapped and placed in a vertical position. The number of flies that crossed the 15 cm mark in 30 s were counted. Three trials were conducted on each set of 20 flies. The data were expressed as the percentage of flies that crossed 15 cm mark.

### Larval olfactory behaviour

The olfactory test was carried out by employing the previous method with minor modifications (as referred in^20^). Larvae were briefly dried on a filter paper before being placed at the centre of the Petri dish. The Petri dish containing 20 µL of Quinine sulphate dispensed on each of the 2 0.5 cm radius filter discs were placed in the diametrically opposite position to Quinine zones. After 2 min of placing the larvae and covering the petri dish, the numbers of larvae in different zones were counted to calculate the percentage of larvae avoiding the bad odour after training.

### Statistical analysis

The statistical analysis was done using unpaired Student’s *t-test*. Untransformed (raw) data were analyzed and plotted by SigmaPlot 10.0 software. The statistical analysis were represented as *, ** and *** where *p < 0.05, **p < 0.001, ***p < 0.0001.

## Supplementary information

Supplementary information

## Data Availability

All the data generated during various experiments are available from the authors on reasonable request.

## Abbreviations

AD: Alzheimer's disease
BCA: Bicinchoninic acid
BSA: Bovine serum albumin
DMSO: Dimethyl sulfoxide
MB: Methylene blue
MTT: Methylthiazolyldiphenyl-tetrazolium bromide
NFTs: Neurofibrillary tangles
PDT: Photodynamic treatment
PHFs: Paired helical filaments
RB: Rose Bengal
SDS–PAGE: Sodium dodecyl sulphate–polyacrylamide gel electrophoresis
SEC: Size-exclusion chromatography
ThS: Thioflavin S
TEM: Transmission electron microscopy
