# Author Correction: Photodynamic exposure of Rose-Bengal inhibits Tau aggregation and modulates cytoskeletal network in neuronal cells

**DOI:** 10.1038/s41598-022-12514-9

**Published:** 2022-06-09

**Authors:** Tushar Dubey, Nalini Vijay Gorantla, Kagepura Thammaiah Chandrashekara, Subashchandrabose Chinnathambi

**Affiliations:** 1 Neurobiology Group, Division of Biochemical Sciences, CSIR-National Chemical Laboratory (CSIR-NCL), Dr. Homi Bhabha Road, Pune, 411008 India; 2 Academy of Scientific and Innovative Research (AcSIR), Ghaziabad, 201002 India; 3 Institution of Excellence, Vijnana Bhavan, University of Mysore, Manasagangotri, Mysore, 570006 India

Correction to: *Scientific Reports* 10.1038/s41598-020-69403-2, published online 23 July 2020

The original version of this Article contained an error in Affiliation 2, which was incorrectly given as ‘Academy of Scientific and Innovative Research (AcSIR), New Delhi 110025, India’. The correct affiliation is listed below:

Academy of Scientific and Innovative Research (AcSIR), Ghaziabad, 201002, India

The original Article has been corrected.

