# Supplementary material for: Photodynamic exposure of Rose-Bengal inhibits Tau aggregation and modulates cytoskeletal network in neuronal cells
Source: Sci Rep. 2020 Jul 23;10:12380. doi: 10.1038/s41598-020-69403-2 (PMC7378248; doi:10.1038/s41598-020-69403-2)
Supplement: Supplementary file 1 — Supplementary information [file 41598_2020_69403_MOESM1_ESM.pdf]

# ESI

## Photodynamic exposure of Rose-Bengal inhibits Tau aggregation and modulates Cytoskeletal network in neuronal cells

**Tushar Dubey[1, 2], Nalini Vijay Gorantla[1, 2] Kagepura Thammaiah Chandrashekara[3], Subashchandrabose Chinnathambi [1, 2*]**

[1]Neurobiology Group, Division of Biochemical Sciences, CSIR-National Chemical Laboratory, Dr. Homi Bhabha Road, 411008 Pune, India

[2] Academy of Scientific and Innovative Research (AcSIR), Ghaziabad, 201002, India

[3]Institution of Excellence, Vijnana Bhavan, University of Mysore, Manasagangotri, 570006 Mysore, India

# Supplementary 1

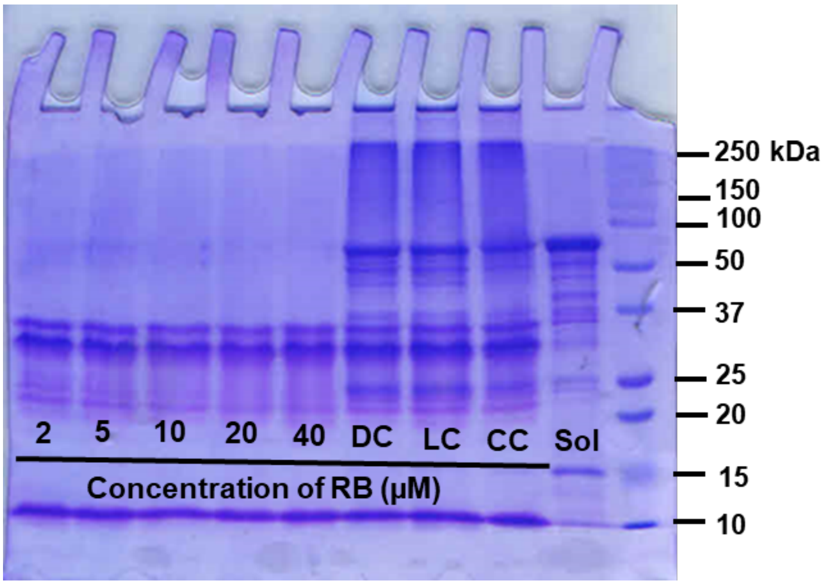


**Supplementary 1: The raw SDS-PAGE image for PE-Rose Bengal Disaggregates Tau filaments.** The aggregates have been treated with various concentrations of photo-excited RB. DC, is designated for "Dark control" where, the aggregates were incubated with 20 µM of non photo-excited RB. LC, designated for "light control" where, the aggregates were exposed to light in absence of RB. CC, is designated for "untreated control". Monomeric Tau was also been loaded on gel for reference which has been designated as "Sol".
